# Comprehensively durable superhydrophobic metallic hierarchical surfaces *via* tunable micro-cone design to protect functional nanostructures†

**DOI:** 10.1039/c7ra13496g

**Published:** 2018-02-12

**Authors:** Jinpeng Han, Mingyong Cai, Yi Lin, Weijian Liu, Xiao Luo, Hongjun Zhang, Kaiyang Wang, Minlin Zhong

**Affiliations:** Laser Materials Processing Research Center, School of Materials Science and Engineering, Tsinghua University Beijing 100084 P. R. China zhml@tsinghua.edu.cn

## Abstract

Superhydrophobic surfaces have been intensively investigated in recent years. However, their durability remains a major challenge before superhydrophobic surfaces can be employed in practice. Although various works have focused on overcoming this bottleneck, no single surface has ever been able to achieve the comprehensive durability (including tangential abrasion durability, dynamic impact durability and adhesive durability) required by stringent industrial requirements. Within the hierarchical structures developed for superhydrophobicity in typical plants or animals by natural evolution, microstructures usually provide mechanical stability, strength and flexibility to protect functional nanostructures to enable high durability. However, this mechanism for achieving high durability is rarely studied or reported. We employed an ultrafast laser to fabricate micro/nanohierarchical structures on metal surfaces with tunable micro-cones and produced abundant nanostructures. We then systematically investigated their comprehensive mechanical durability by fully utilizing the protective effect of the microstructures on the functional nanostructures *via* the tunable design of micro-cones. We confirm that the height and spatial period of the microstructures were crucial for the tangential abrasion durability and dynamic impact durability, respectively. We finally fabricated optimized superhydrophobic tungsten hierarchical surfaces, which could withstand 70 abrasion cycles, 28 min of solid particle impact or 500 tape peeling cycles to retain contact angles of greater than 150° and sliding angles of less than 20°, which demonstrated exceptional comprehensive durability. The comprehensive durability, in particular the dynamic impact durability and adhesive durability, are among the best published results. This research clarifies the mechanism whereby the microstructures effectively protected the functional nanostructures to achieve high durability of the superhydrophobic surfaces and is promising for improving the durability of superhydrophobic surfaces and thus for practical applications.

## Abstract 2

Superhydrophobic metallic hierarchical surfaces with micro-cones designed to protect functional nanostructures exhibit outstanding comprehensive mechanical durability.

## Graphical abstract

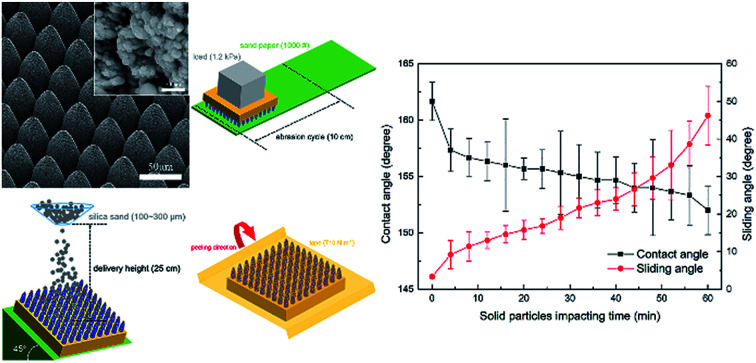

## Introduction

Superhydrophobic surfaces have gained increasing attention owing to their wide range of potential applications in the areas of self-cleaning,^1–3^ anti-icing,^4–6^ anticorrosion,^7–11^ oil–water separation,^12,13^*etc.* However, one problem needs thorough resolution before superhydrophobic surfaces are widely employed in industry or daily life, namely, durability.^14–16^ When superhydrophobic surfaces are employed in practice, they must be able to withstand complicated environmental stimuli, *e.g.*, mechanical damage, UV exposure, chemical reactions, bacterial contamination, *etc.*^14–16^ Among these kinds of stimuli, mechanical damage is the most common and frequent challenge and thus becomes the main factor leading to the failure of superhydrophobic surfaces. Common forms of mechanical damage include wearing, impacting, and peeling, which correspond to tangential abrasion durability, dynamic impact durability and adhesive durability, respectively.^15^ When superhydrophobic surfaces undergo mechanical damage, their surface hydrophobic components may be lost and/or their functional hierarchical structures may be destroyed. Both cases lead to a decline in superhydrophobicity. During the loss of superhydrophobicity, the sliding angle is the most sensitive indicator and increases rapidly, whereas the contact angle always decreases slowly at first.^15^ Obviously, it is vital to improve the mechanical durability of superhydrophobic surfaces.

Typically, there are three routes for producing superhydrophobic surfaces, namely, hydrophobic coatings,^17,18^ hydrophobic bulk materials,^19–23^ and micro/nanohierarchical structures with surface hydrophobic modifications.^24–28^ Hierarchical structures can be observed widely in nature as the results of billions of years of evolution, such as lotus leaves, fish scales, butterfly wings, spider silk, *etc.*^29^ Within these hierarchical structures developed by natural evolution, it is well understood that the nanostructures provide the main functionalities, such as wettability, directional adhesion, antireflective properties, selective filtration, *etc.*, whereas the microstructures provide the mechanical stability, strength and flexibility responsible for supporting and protecting the functional nanostructures.^29^ Thus, superhydrophobic surfaces with micro/nanohierarchical structures are promising for achieving both functional superhydrophobicity and mechanical durability.

After superhydrophobic surfaces have been intensively investigated in recent years, the significance of improving their mechanical durability is apparently appealing to researchers around the world, as many studies have been performed so far.^4,8,11,30–47^ Liu *et al.* produced superhydrophobic surfaces on X80 pipeline steel substrates by the electrochemical deposition of copper and subsequent chemical bath processing. The resulting CuO flower-like hierarchical laminated structures endowed the superhydrophobic surfaces with durability. After 10 abrasion cycles, the contact angles decreased from 163° to 150°, whereas a change in the sliding angles was not reported.^32^ Zhang *et al.* fabricated superhydrophobic surfaces by the one-step spray coating of hydrophobic nanoparticles on an etched aluminum alloy with pitted morphology. Both the tangential abrasion durability and the dynamic impact durability were enhanced by the cushion effect, *i.e.*, the nanoparticles could be embedded within protrusions, on sidewalls or between grooves to provide protection. The sliding angles were less than 60° and 20° after 5 abrasion cycles and 5 solid particle impact cycles, respectively.^39^ Barthwal *et al.* produced superhydrophobic aluminum hierarchical surfaces by chemical etching and anodization. Owing to the downward-directed features formed by nanopores on the microtextures, the surfaces could sustain 10 tape peeling cycles without a decrease in contact angles, although data for the sliding angles were lacking.^40^ In addition to the above research, some review papers have also been published recently that reported progress in the production of mechanical durable superhydrophobic surfaces.^14–16^ In 2016, Milionis *et al.* published a review paper on mechanically durable superhydrophobic surfaces by summarizing the exponentially increasing publications on this topic. After describing in detail the various works and progress in improving the mechanical durability of superhydrophobic surfaces, they indicated that no single surface had ever been able to pass all the types of durability tests required by stringent industrial requirements and standards for commercialization. They then recommended that measurements of sliding angles need to be performed, because these are more important for functionality and are the most likely to be affected in durability tests.^15^ On the basis of the above analysis, it is recognized that the challenge of achieving mechanical durability of superhydrophobic surfaces has still not been adequately overcome, as is mainly indicated by the following four aspects. Firstly, many published papers report data for contact angles, whereas more important data for sliding angles are sometimes ignored, which inevitably creates a barrier to the comparison of the durability of the superhydrophobic surfaces reported by various research groups, as indicated by Milionis *et al.*^15^ Secondly, so far published papers have usually reported a single durability of superhydrophobic surfaces, but few papers have ever tried to completely cover the comprehensive durability of superhydrophobic surfaces, *i.e.*, tangential abrasion durability, dynamic impact durability and adhesive durability, at the same time. Thirdly, current superhydrophobic surfaces are typically produced by chemical methods, whereby the hierarchical structures are usually fragile and the microstructures are difficult to control. Lastly, in natural hierarchical structures, the microstructures usually provide the mechanical strength and stability to protect the vulnerable but functional nanostructures. This mechanism for achieving high durability is rarely studied or reported.

In this study, we employed an ultrafast laser to fabricate and/or produce micro/nanohierarchical structures on metal surfaces. By modulating the processing strategy and the parameters of the ultrafast laser, we were able to produce micro-cones with tunable heights and spatial periods on various kinds of metal surfaces. At the same time, abundant nanostructures were produced on the outer surface of these micro-cones. This enabled us to obtain excellent superhydrophobic surfaces with contact angles of up to 163° and sliding angles of as little as 2°. We then systematically investigated their comprehensive durability, including their tangential abrasion durability, dynamic impact durability and adhesive durability, by fully utilizing the protective effect of the microstructures on the functional nanostructures *via* the tunable design of the micro-cones. The influence of the height and spatial period of the microstructures on the mechanical durability of the superhydrophobic metallic hierarchical surfaces was investigated, and the loss of superhydrophobicity was also explained in detail. By optimizing the protective microstructures on the nanostructures, we finally fabricated optimized superhydrophobic tungsten hierarchical surfaces, which could withstand 70 abrasion cycles in linear abrasion tests, 28 min of solid particle impact in solid particle impact tests and 500 tape peeling cycles in tape peeling tests. After these tests, the contact angles remained greater than 150° and the sliding angles less than 20°, which demonstrated exceptional comprehensive durability. The comprehensive durability that was achieved, in particular the dynamic impact durability and adhesive durability, is among the best published results to the best of our knowledge. Our research clarifies the mechanism whereby the microstructures effectively protected the functional nanostructures to achieve high durability of the superhydrophobic surfaces, as occurs in nature, which is promising for enhancing the durability of superhydrophobic surfaces for practical applications.

## Experimental

### Preparation of superhydrophobic metallic hierarchical surfaces

Metallic hierarchical surfaces were fabricated using a Trumpf TruMicro 5000 femtosecond laser. Its pulse width, repetition rate and maximum average power were 800 fs, 200 kHz and 40 W, respectively. Pristine metal plates with dimensions of 10 × 10 × 2 mm were polished with sandpaper and then rinsed ultrasonically with ethanol before laser processing. The focused laser beam scanned the surfaces of the metal plates in a pattern of intersecting lines under an ambient environment using an x-y galvo scanning system and an f-θ lens. The laser processing parameters were a laser power of 5 W (for sample W) or 10 W, scanning speeds of 50 mm s^−1^ (for sample NS) or 500 mm s^−1^, scanning intervals of 10 μm (for sample NS), 40 μm, 60 μm, 80 μm, 100 μm or 120 μm, and scanning times of 1 (for sample NS), 8, 14, 18, 30 or 60. After laser processing, the samples were immersed in a solution in isopropyl alcohol of perfluorodecyltrimethoxysilane with a mass fraction of 0.5% for 2 h and then dried at 80 °C for 2 h.

### Characterization

The surface morphologies of the samples were examined using field-emission scanning electron microscopy (SEM, FEI Quanta 200 FEG). The 3D microstructures of the samples were measured with a laser scanning confocal microscope (Olympus LEXT OLS4100). The chemical states of elements were analyzed *via* X-ray photoelectron spectroscopy (XPS). The distribution of elements was analyzed by energy-dispersive spectroscopy (EDS, Oxford). Measurements of the wettability of the samples were performed by measuring the contact angles (CAs) using the sessile drop technique *via* a video-based optical contact angle measuring device (OCA 15 Plus, Data Physics Instruments). The sliding behavior of water droplets on the samples was investigated by measuring the sliding angles (SAs) using the tilting plate method. The volumes of the water droplets selected for the CA and SA measurements were 5 μL. Each CA or SA test was repeated three times, and the average result was used.

### Durability tests

Linear abrasion tests, solid particle impact tests and tape peeling tests were performed to assess the tangential abrasion durability, dynamic impact durability and adhesive durability of the superhydrophobic surfaces, respectively. In the linear abrasion tests, 1000# sandpaper was selected as an abrasive surface, and the superhydrophobic surfaces were placed facing the abrasive surface with a load of 1.2 kPa. An abrasion cycle was defined as a relative motion of 10 cm back and forth (see Fig. 3a). In the solid particle impact tests, the superhydrophobic surfaces were fixed on a substrate, which was tilted at 45°. Silica sand with diameters ranging from 100 to 300 μm was released from a height of 25 cm to fall on the superhydrophobic surfaces. The rate of silica sand flow was kept at 10 g min^−1^ (see Fig. 4a). In the tape peeling tests, 3M 250# test tape with an adhesive strength of 710 N m^−1^ was used as received. A tape peeling cycle was defined as an entire process comprising three steps. Firstly, the tape was applied to the superhydrophobic surface. Secondly, the tape was pressed to ensure good contact with the superhydrophobic surface. Thirdly, the tape was peeled from one end.

## Results and discussion

### Fabrication of superhydrophobic metallic hierarchical surfaces

**Fig. 1:**
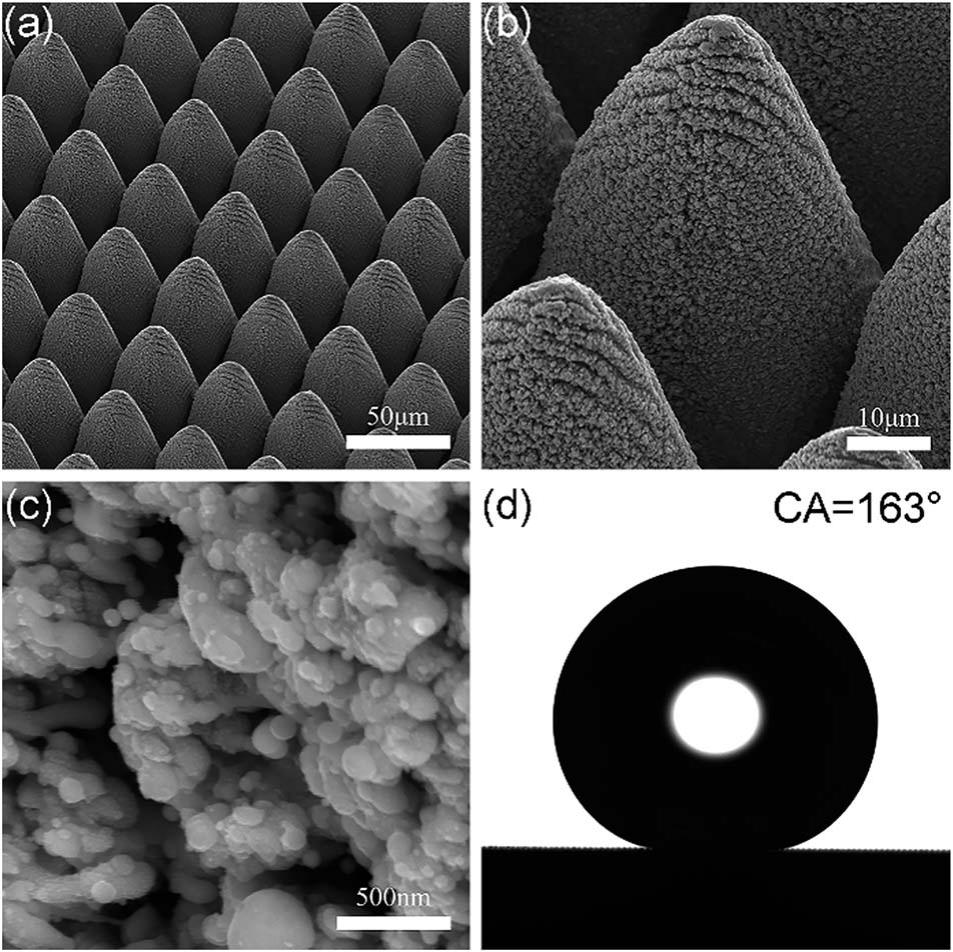
Surface morphology and non-wetting property of the Cu-based superhydrophobic hierarchical surfaces. (a)–(c) SEM images at different magnifications. (d) Contact angle with a water drop of 5 μL.

A typical ultrafast-laser-based top-down strategy was used to fabricate the superhydrophobic metallic hierarchical surfaces. Metallic hierarchical surfaces were obtained *via* ultrafast laser ablation, as described in detail in the experimental section. Ultrafast ablation is a universal micro/nanostructuring method without limitations as to materials. We selected copper as an initial material to study the mechanical durability of the superhydrophobic metallic hierarchical surfaces for the reasons that copper is a common widely used material in industry and daily life and is a relatively soft metal, and the influence of mechanical damage on superhydrophobic surfaces with different surface structures could therefore be magnified. Using an optimized laser processing strategy and laser scanning parameters, copper-based micro/nanohierarchical surfaces were obtained, as shown in Fig. 1a–c. On the microscale, an ordered array of micro-cones was formed after femtosecond laser ablation; the spatial period of the micro-cone array was 40 μm and the height of each micro-cone was 60 μm. In contrast to traditional lasers, a femtosecond laser has a pulse duration of 800 fs, which is obviously shorter than the phonon–electron coupling time (typically around 10 picoseconds). Therefore, femtosecond laser processing is a typical ablation process instead of a melting process, with a negligible heat-affected zone and high processing precision. On the nanoscale, densely distributed nanoparticle clusters appeared on each micro-cone. The diameters of most particles were around 100 nm, and the cluster combination displayed porous features. The formation of the nanoparticle clusters was mainly due to the falling back of the plasma plume with a relatively low initial kinetic energy, which was induced by the interaction of the high-fluence ultrafast laser and the metal surfaces. It is worth noting that ultrafast laser ablation is a one-step, maskless, simple, green and efficient method that is economical for large-scale applications in industry. The fabrication of a hierarchical surface with dimensions of 10 × 10 mm took just less than 8 min.

After the metallic hierarchical surfaces were formed by ultrafast laser ablation, surface chemical modification with a fluorinating agent, namely, perfluorodecyltrimethoxysilane, was performed on the surfaces to achieve water repellency performance. The F 1s XPS spectra in Fig. S1† prove the successful fluorination of the as-prepared hierarchical structures. After fluorination, the metallic hierarchical surfaces displayed a typical Cassie state with a water contact angle of up to 163° and a sliding angle of as little as 2°, as shown in Fig. 1d and Fig. S1†. In addition to the low surface free energy achieved by fluorination, the high micro/nanoscale roughness due to the well-designed micro-cone array and the abundant nanoparticles that were produced contributed greatly to the excellent superhydrophobicity. In particular, the porous features of the nanoparticle clusters further increased the surface roughness and at the same time allowed the self-assembly of perfluorodecyltrimethoxysilane molecules inside the porous nanoparticle clusters. This led to the excellent superhydrophobicity by providing enough room for “air traps” beneath a water drop to form a typical Cassie state.

### Influence of micro-cone height on the mechanical durability of superhydrophobic metallic hierarchical surfaces

**Fig. 2:**
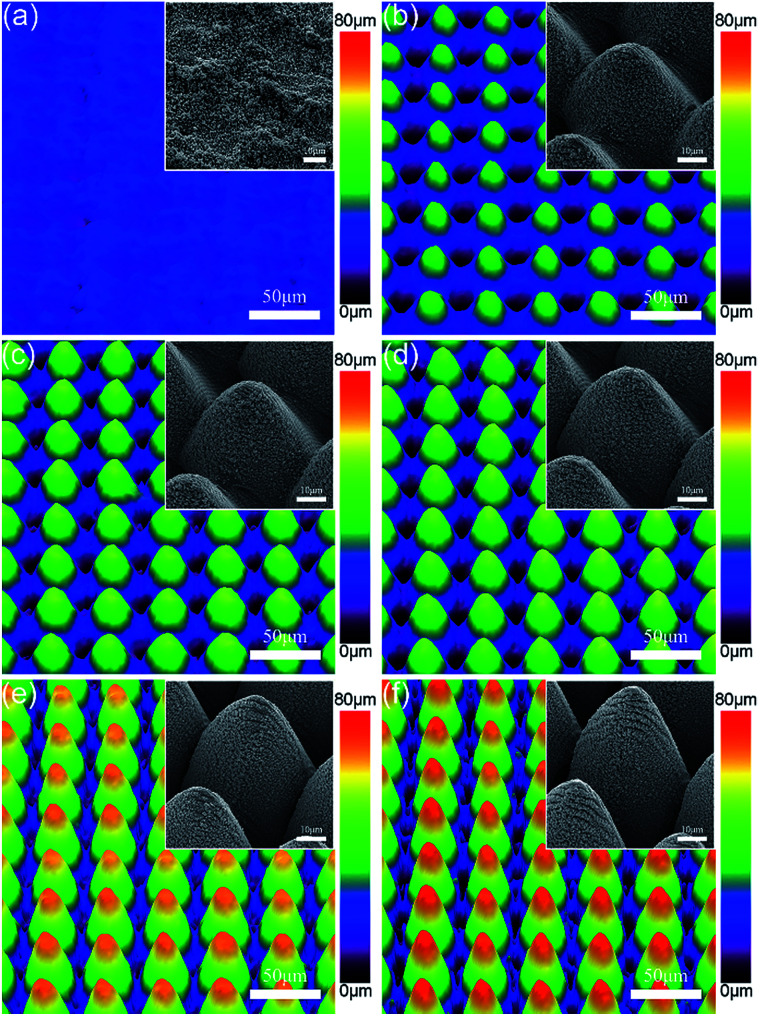
Laser scanning confocal microscopy images and SEM images of (a) sample NS, (b) sample H25, (c) sample H35, (d) sample H40, (e) sample H50 and (f) sample H60.

In comparison with superhydrophobic metallic surfaces with single-scale nanostructures, superhydrophobic metallic hierarchical surfaces are theoretically supposed to exhibit higher mechanical durability owing to the protective effect of the stronger microstructures on the fragile functional nanostructures, as commonly appears in nature. However, this mechanism for achieving high durability has rarely been studied or reported to date. Thanks to the high capability of ultrafast laser ablation to form various microscale structures, we were able to design and produce diverse micro-cone structures with tunable micro-cone heights and spatial periods. We could then systematically investigate the protective effect of the micro-cones on the nanostructures for improving the mechanical durability of the metallic superhydrophobic surfaces. Both the micro-cone height and the spatial period could be tailored by merely tuning the laser processing strategy and laser scanning time. Fig. 2b–f illustrates superhydrophobic metallic hierarchical surfaces with the same micro-cone period of 40 μm but tuned micro-cone heights of 25 μm (named as sample H25), 35 μm (named as sample H35), 40 μm (named as sample H40), 50 μm (named as sample H50) and 60 μm (named as sample H60), respectively. For comparison and reference, a superhydrophobic metallic surface with single-scale nanostructures (named as sample NS) was produced (see Fig. 2a). From the insets, it can be observed that despite the obvious differences in the micro-cone heights, the six samples were similar on the nanoscale. Each of these six superhydrophobic surfaces exhibited a contact angle of greater than 155° and a sliding angle of less than 7°, which indicated a good Cassie state.

**Fig. 3:**
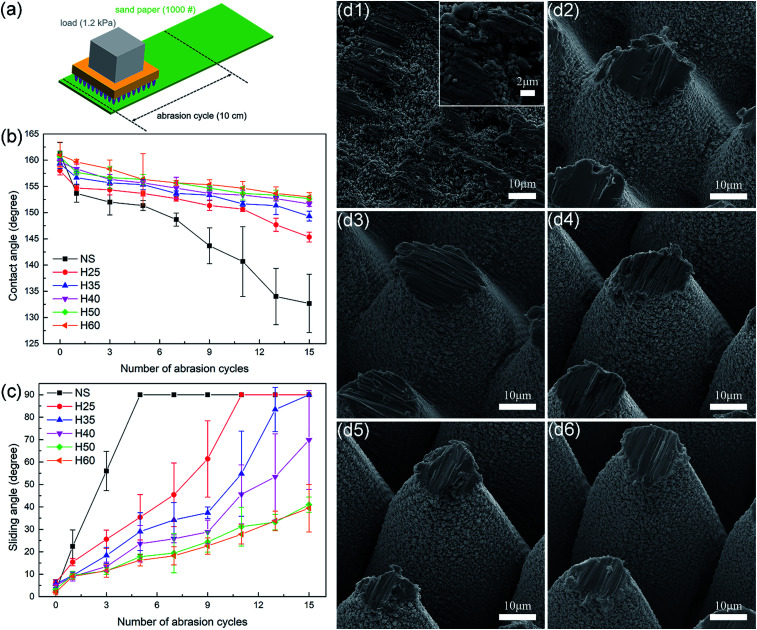
(a) Schematic of linear abrasion tests. (b) Contact angle and (c) sliding angle as a function of the number of abrasion cycles. (d) SEM images of (d1) sample NS, (d2) sample H25, (d3) sample H35, (d4) sample H40, (d5) sample H50 and (d6) sample H60 after 15 abrasion cycles.

Mechanical durability typically includes adhesive durability, tangential abrasion durability and dynamic impact durability. Tangential abrasion durability is one of the most important aspects of mechanical durability, because abrasion is so common in applications that it is basically the first reason that leads to the failure of superhydrophobic surfaces. The usual methods for measuring tangential abrasion durability include the linear abrasion test, circular abrasion test, blade/knife test, pencil hardness test, *etc.*, among which the linear abrasion test is the most widely accepted.^15^Fig. 3a shows a simple schematic of the linear abrasion test, in which 1000# sandpaper was selected as the abradant, which provided more severe abrasion conditions than other abradants such as cloth, rubber, *etc.*

Fig. 3b and c compares the durability performance of superhydrophobic metallic hierarchical surfaces with different micro-cone heights. In the absence of any protection by microstructures, sample NS obviously performed much worse than those with hierarchical surfaces. The black lines for sample NS in Fig. 3b and c show a complete loss of superhydrophobicity under mechanical damage, which included two main stages. In the first stage, or the “SA varying stage”, the sliding angles increased rapidly from 2.6° to 90° during the first five abrasion cycles while the contact angles remained larger than 150°, which indicated a complete transition from the Cassie state to the Wenzel state. In the second stage, or the “CA varying stage”, as the abrasion cycles continued to increase the sliding angles remained at 90°, while the contact angles decreased gradually and finally reached 133° after 15 abrasion cycles. Fig. 3d shows the surface morphology of sample NS after 15 abrasion cycles. It is obvious that without the protection of the microstructures the functional nanoparticles were completely destroyed on a large scale, in particular when compared with the original surface morphology shown in Fig. S2†. This destruction left a quite large defect area where water drops would adhere, which led to the failure of superhydrophobicity.

In comparison with sample NS, the samples with hierarchical surfaces displayed higher tangential abrasion durability. As the height of the micro-cones increased, the tangential abrasion durability improved accordingly. sample H25 and sample H35 entered the CA varying stage after 11 and 15 abrasion cycles, respectively. In contrast, the samples with greater micro-cone heights remained in the SA varying stage after 15 abrasion cycles, with sliding angles of 69.8°, 41° and 39.4° for sample H40, sample H50 and sample H60, respectively. It should be noted that sample H50 and sample H60 performed similarly well in the linear abrasion tests. From Fig. 3d, it can be observed that with the protection of the micro-cones, nanoparticles on the sidewalls of micro-cones were preserved well in the linear abrasion tests, while the height of each sample decreased as the tops of the micro-cones were worn off. This indicates that the heights of hierarchical surfaces must exceed a certain value to sustain a Cassie state. With an increase in the number of abrasion cycles, samples with lower microscale heights are more likely to be below the threshold and lose superhydrophobicity than those with greater microscale heights. In addition, samples with greater micro-cone heights had smaller defect areas than those with lower micro-cone heights under the same abrasion conditions.

**Fig. 4:**
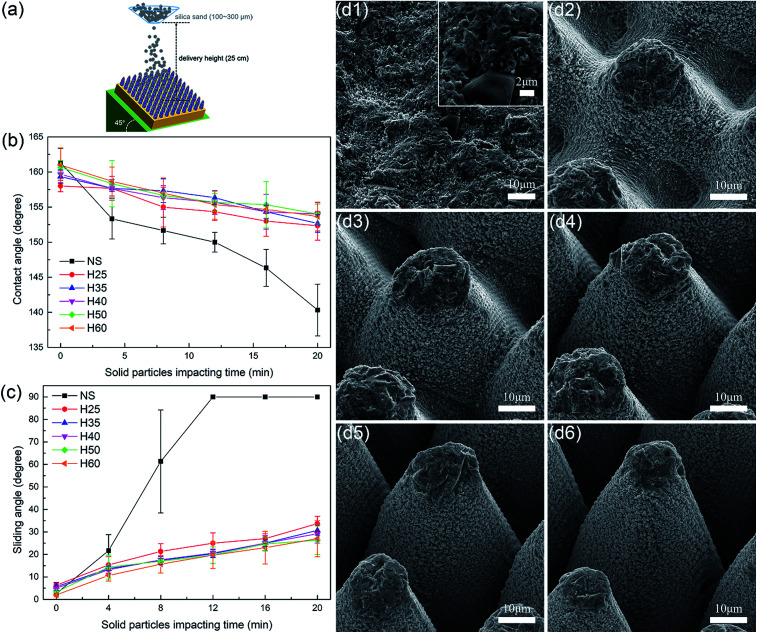
(a) Schematic of solid particle impact test. (b) Contact angle and (c) sliding angle as a function of the solid particle impact time. (d) SEM images of (d1) sample NS, (d2) sample H25, (d3) sample H35, (d4) sample H40, (d5) sample H50 and (d6) sample H60 after 20 min of solid particle impact.

Besides tangential abrasion durability, dynamic impact durability is also crucial for superhydrophobic surfaces in outdoor applications owing to the common harsh conditions outside. The solid particle impact test, liquid spray/jet/droplet impact test and aerodynamic impact test can be adapted to measure the dynamic impact durability of superhydrophobic surfaces, among which the solid particle impact test is common and easy to control.^15^Fig. 4a schematically illustrates the process of the solid particle impact test. When the silica sand particles reach the surface, their kinetic energy is absorbed by the surface, which leads to the destruction of the surface.

The protective effect of the micro-cones played a predominant role in the solid particle impact tests, as can be seen in Fig. 4b and c. Sample NS entered the CA varying stage after 12 min of solid particle impact, while the samples with hierarchical surfaces still remained in the “SA varying stage” even after 20 min of solid particle impact. The height of the micro-cones played a minor role in the solid particle impact tests. Sample H50 and sample H60 performed the best, with sliding angles of 26.3° and 27°, which were only a few degrees less than that of sample H25, *i.e.*, 33.7°. Fig. 4d shows the surface morphologies of all six samples after 20 min of solid particle impact. The functional nanoparticles of sample NS were severely damaged in comparison with the original morphology shown in Fig. S2† as a result of the lack of protection by micro-cones. In the samples with hierarchical surfaces, the nanoparticles remained well preserved except for those near the tops of micro-cones, which only constituted a small proportion of all the nanostructures. Notably, the height of the micro-cones did not decrease markedly in the solid particle impact tests, as the damage arose from impacts rather than abrasion, which also explains the imperceptible differences between samples with different heights.

In conclusion, with the protection of the micro-cones, both the tangential abrasion durability and the dynamic impact durability of the superhydrophobic metallic hierarchical surfaces were much higher than those of superhydrophobic metallic surfaces with single-scale nanostructures. As the height of the micro-cones increased, the tangential abrasion durability of the superhydrophobic metallic hierarchical surfaces improved, but the improvement became negligible after the micro-cones reached a certain height. In terms of dynamic impact durability, the superhydrophobic metallic hierarchical surfaces with greater micro-cone heights performed better, but the differences were not obvious. Therefore, the height of the micro-cones played a more important role in the improvement of the tangential abrasion durability than in the dynamic impact durability. In order to achieve higher comprehensive mechanical durability, it is advised that relatively higher micro-cones are needed for superhydrophobic metallic hierarchical surfaces.

### Influence of the micro-cone spatial period on the mechanical durability of superhydrophobic metallic hierarchical surfaces

**Fig. 5:**
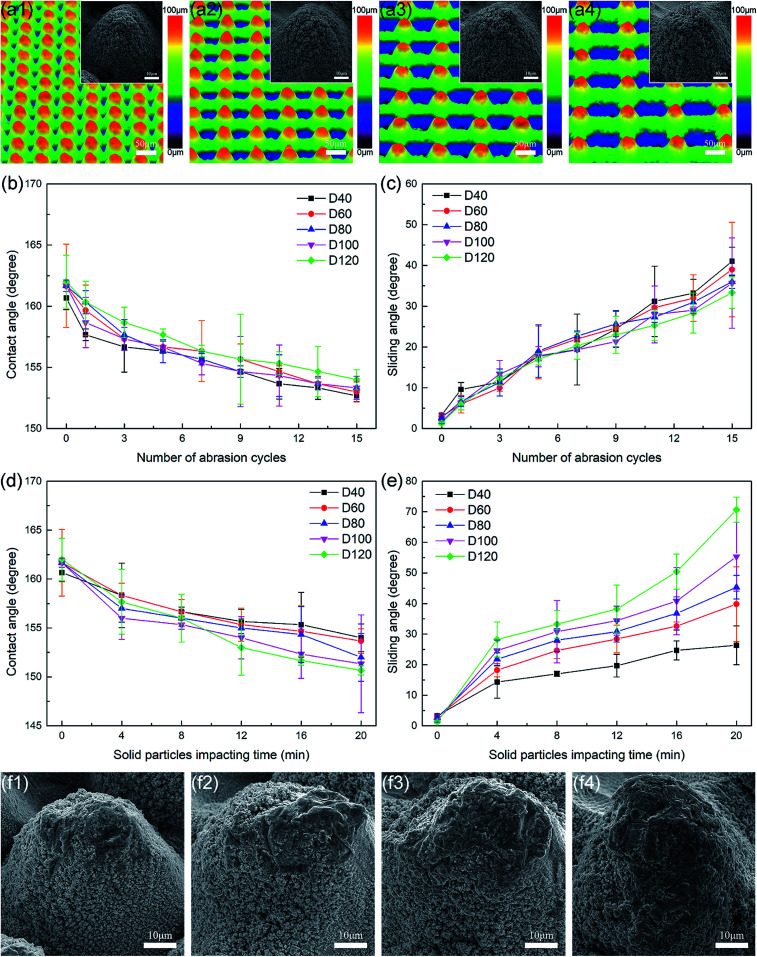
(a) Laser confocal microscopy images and SEM images of (a1) sample D60, (a2) sample D80, (a3) sample D100, and (a4) sample D120. (b) Contact angle and (c) sliding angle as a function of the number of abrasion cycles. (d) Contact angle and (e) sliding angle as a function of the solid particle impact time. (f) SEM images of (f1) sample D60, (f2) sample D80, (f3) sample D100, and (f4) sample D120 after 20 min of solid particle impact.

Because the height and spatial period of the micro-cones are both key parameters that define a micro-cone array, it is necessary to study the role that the micro-cone spatial period plays in affecting the tangential abrasion durability and the dynamic impact durability of superhydrophobic metallic hierarchical surfaces. By simply tuning the laser scanning pattern in the femtosecond laser ablation process, superhydrophobic metallic hierarchical surfaces with tailored periods were fabricated. Fig. 5a shows as-prepared superhydrophobic metallic hierarchical surfaces with the same micro-cone height of 50 μm but tuned periods of 60 μm (named as sample D60), 80 μm (named as sample D80), 100 μm (named as sample D100) and 120 μm (named as sample D120), respectively. Together with sample H50, which had the same height but a period of 40 μm (see Fig. 2e; sample H50 is also named as sample D40 here), five samples in total were tested systematically for their tangential abrasion durability and dynamic impact durability.

The influence of the micro-cone period on the tangential abrasion durability of the superhydrophobic metallic hierarchical surfaces is shown in Fig. 5b and c. Despite the great difference in their periods, the five samples differed little in the linear abrasion tests. After 15 abrasion cycles, all the samples remained in the SA varying stage. The sliding angles decreased by only a few degrees with an increase in the period; sample D40 had the largest sliding angle of 41° and sample D120 had the smallest sliding angle of 33.3°. From Fig. 3d and S3† it can be observed that the single micro-cones in all five samples had similar defect areas and were of similar heights after 15 abrasion cycles. However, with an increase in the micro-cone period the micro-cones became sparser, as did the defect areas, which led to a slightly higher tangential abrasion durability.

However, with respect to the dynamic impact durability, the influence of the micro-cone period became significant. Fig. 5d and e clearly demonstrates that the dynamic impact durability gradually deteriorated as the period increased. After 20 min of solid particle impact, although all five samples remained in the SA stage, the sliding angles varied distinctly from 26.3° for sample D40 to 70.7° for sample D120. This phenomenon could be explained by an analysis of Fig. 4d and 5f. On comparing the surface morphologies of all five samples after 20 min of solid particle impact, the defect areas apparently became larger with an increase in the period. Considering that the solid particles used were SiO_2_ particles with diameters ranging from 100 μm to 300 μm, it is reasonable to estimate that with an increase in the period the SiO_2_ particles were more likely to intrude into the space between two neighboring micro-cones, contact the sidewalls and thus cause a larger defect area. For sample D40 and sample D60, the defect areas were mainly distributed on the tops of the micro-cones as a result of a “surface contact only” situation. However, for the samples with larger periods the defect areas extended to the sidewalls of the micro-cones, which indicated an “intruding contact” situation. In particular for sample D120, of which the period was even greater than that of some of the SiO_2_ particles, the defect area extended along the sidewall almost to the bottom. This large defect area explains the disappointing dynamic impact durability of sample D120.

In conclusion, the spatial period of the micro-cones had a more significant influence on the dynamic impact durability than on the tangential abrasion durability of the superhydrophobic metallic hierarchical surfaces. With an increase in the period, the tangential abrasion durability improved slightly, but the dynamic impact durability deteriorated noticeably. Therefore, higher comprehensive mechanical durability of superhydrophobic metallic hierarchical surfaces requires a relatively small spatial period of the micro-cones. Considering that the beam waist of the femtosecond laser that was used was around 38 μm, 40 μm is basically the smallest spatial period that guarantees a uniform micro-cone array and is thus the optimized micro-cone spatial period.

### Discussion of loss of superhydrophobicity from the viewpoint of the loss of the surface hydrophobic component

Usually, there are two reasons for the loss of the superhydrophobicity of a non-wetting surface under mechanical damage, namely, the destruction of the surface hierarchical morphology and the loss of surface hydrophobic components. In the above linear abrasion tests and solid particle impact tests, the results mainly report the loss of superhydrophobicity from the viewpoint of the surface morphology. However, the loss of the surface hydrophobic component on the as-prepared superhydrophobic metallic hierarchical surfaces, *i.e.*, the molecular layers of perfluorodecyltrimethoxysilane, is also an important reason for the loss of superhydrophobicity and needs to be investigated carefully.

**Fig. 6:**
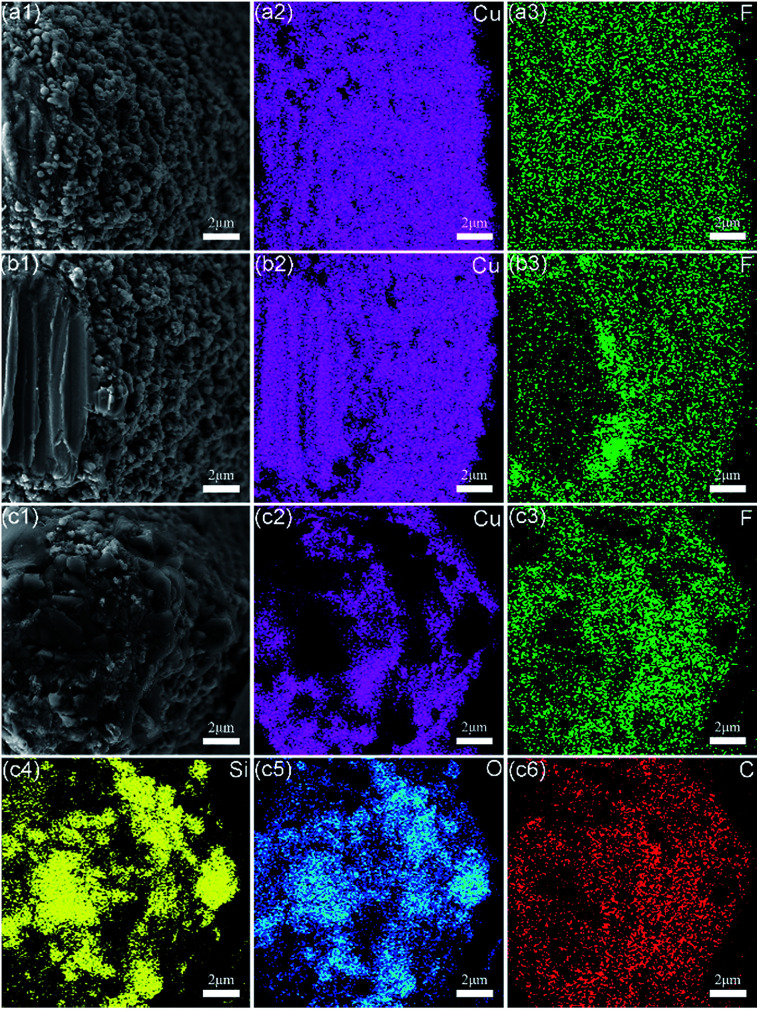
Results of EDS mapping analysis of sample H25 (a) before mechanical durability tests, (b) after 15 abrasion cycles, and (c) after 20 min of solid particle impact.

Sample H25 was selected for EDS mapping analysis as an example. Fig. 6a shows the results of elemental analysis of Cu and F before the sample underwent any durability tests. It can be seen that both Cu element and F element display relatively uniform distributions, which indicates an ideal fluorination process by perfluorodecyltrimethoxysilane. After 15 abrasion cycles in the linear abrasion tests (see Fig. 6b), the top of the micro-cone was worn off into a relatively flat defect area, and the copper substrate underneath appeared to be the new surface. Therefore, the distribution of Cu element remained relatively uniform. However, F element exhibited an obviously heterogeneous distribution. The newly appearing defect area contained much less F element than previously, which indicated that the defect area lost the original hydrophobic component in the linear abrasion tests. Regarding the sidewall of the micro-cone, the distribution of F element remained unchanged as a result of the protective effect of the micro-cones.

Fig. 6c shows the results of elemental analysis of sample H25 after 20 min of solid particle impact in the solid particle impact tests. Both Cu element and F element had heterogeneous distributions. However, Si element and O element were abundant where Cu element and F element were lacking, which indicated the presence of debris from the SiO_2_ particles in these zones. In the SEM images in Fig. 6c, some dark and sharp polyhedron-like debris can be clearly observed. The debris particles had diameters ranging from 1 μm to 5 μm and were located exactly where Si element and O element were abundant. These results match well and prove that in the solid particle impact tests some debris from the SiO_2_ particles appeared during the impacting process and became embedded in some zones of the surface of the micro-cone to form so-called “defect areas”. Unlike the situation in the linear abrasion tests, the defect areas formed in the solid particle impact tests were discontiguous, so that even on the top of the micro-cone F element was partly visible. Therefore, from the viewpoint of the loss of the surface hydrophobic component, solid particle impact tests are milder than linear abrasion tests.

### Comprehensive durability of tungsten-based superhydrophobic metallic hierarchical surfaces with optimized microstructures

From the above results and discussion, it is now clear that a relatively greater micro-cone height and a relatively smaller micro-cone spatial period are beneficial for the improvement of both tangential abrasion durability and dynamic impact durability. In addition, the intrinsic mechanical properties of metallic materials are other important factors that affect the comprehensive durability. Relatively harder metallic materials such as tungsten, titanium, *etc.* are obviously less likely to undergo wear than softer metallic materials such as copper, nickel, *etc.* under the same conditions. In order to obtain superhydrophobic metallic hierarchical surfaces with comprehensive durability, the metallic material itself needs to be considered, in addition to the optimization of the micro-cone height and spatial period.

**Fig. 7:**
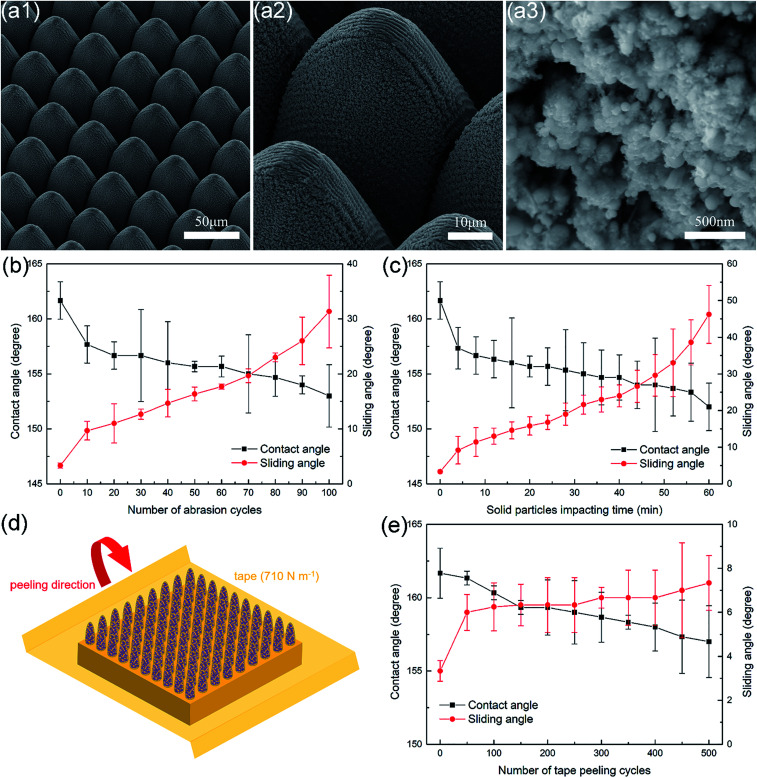
(a) SEM images of tungsten-based superhydrophobic metallic hierarchical surfaces at different magnifications. (b) Contact angle and sliding angle as a function of the number of abrasion cycles. (c) Contact angle and sliding angle as a function of the solid particle impact time. (d) Schematic of tape peeling tests. (e) Contact angle and sliding angle as a function of the number of tape peeling cycles.

Therefore, as an extreme example, tungsten-based superhydrophobic metallic hierarchical surfaces with optimized microstructures (named as sample W) were produced *via* femtosecond laser ablation to achieve outstanding comprehensive durability, as can be seen in Fig. 7a. Sample W was constructed with micro-cone arrays on the microscale and nanoparticle clusters on the nanoscale. The height and spatial period of the micro-cones were 50 μm and 40 μm, respectively. The diameters of the nanoparticles ranged from 10 nm to 100 nm, which were smaller than those of the copper-based superhydrophobic metallic hierarchical surfaces, and they also displayed porous features. The fabrication of the tungsten-based superhydrophobic metallic hierarchical surfaces also proves that ultrafast laser ablation is a universal micro/nanostructuring method that can be applied to a wide range of materials.

Thanks to the optimization of both the micro-cones and the material, the as-prepared tungsten-based superhydrophobic metallic hierarchical surfaces exhibited exceptional comprehensive durability. In linear abrasion tests, the sliding angles of sample W remained less than 20° even after 70 abrasion cycles. After as many as 100 abrasion cycles, sample W still remained in the SA varying stage, with a contact angle of 153° and a sliding angle of as little as 31.3° (see Fig. 7b), which demonstrates high tangential abrasion durability in comparison with superhydrophobic surfaces reported by other groups, considering that one abrasion cycle in this study represented an abrasion distance of 20 cm (see Table S1†).^4,8,11,30,31,34,47^ The high tangential abrasion durability arose from the greater height of the micro-cones made from the intrinsically antiwear material tungsten, which provided ideal protection for nanoparticles on the sidewalls of micro-cones (see Fig. S4†). In terms of dynamic impact durability, sample W performed outstandingly. Its sliding angles remained less than 20° even after 28 min of solid particle impact. After as long as 60 min of solid particle impact, sample W still maintained a contact angle of 152° and a sliding angle of 46.2°, as seen in Fig. 7c. Owing to the dense arrangement of the tungsten micro-cones, the particle impact only damaged a very small zone near the tops of the micro-cones, which left nanoparticles on the sidewalls well preserved (see Fig. S4†). As 28 min of solid particle impact represents the impact of 280 g SiO_2_ particles, the dynamic impact durability of sample W is among the best published results (see Table S1†).^32,35–39,44,46^

Besides, the tungsten-based superhydrophobic metallic hierarchical surfaces also displayed excellent adhesive durability. Adhesive durability is important because the lack of it will cause severe problems, such as the detachment of the hydrophobic component from the substrate, which results in the loss of superhydrophobicity. Methods such as the tape peeling test, cross-cut test, and so on can be carried out to measure the adhesive durability of superhydrophobic surfaces, among which the tape peeling test is the simplest and most effective.^15^Fig. 7d shows a schematic of the tape peeling test. Different tapes have different cohesive adhesions, which are defined as the strength of adhesion to steel in N m^−1^. The tape used in this work was 3M 250# test tape with an adhesive strength of 710 N m^−1^, which is a standard test tape. From Fig. 7e, it can be observed that sample W was able to maintain a sliding angle of less than 10° even after 500 tape peeling cycles, which demonstrates a markedly higher adhesive durability than reported results, as no reported superhydrophobic surfaces could withstand more than 100 tape peeling cycles (see Table S1†).^38,40–44^

In fact, the superhydrophobic metallic hierarchical surfaces fabricated *via* the previously discussed ultrafast laser ablation method all exhibited similar excellent adhesive durability. The performance of sample H25 in tape peeling tests is given in Fig. S5† as an example. In tape peeling tests, the tapes could only contact some of the nanoparticles on the tops of the micro-cones, and most of the nanoparticles present on the sidewalls of the micro-cones were not touched during the tape peeling process and thus remained undamaged. Therefore, even after 500 tape peeling cycles, the surface micro/nano morphology and the surface hydrophobic component were well preserved (see Fig. S5†), which confirmed the excellent adhesive durability.

## Conclusions

In summary, superhydrophobic metallic hierarchical surfaces were successfully produced *via* ultrafast laser ablation. In order to study the protective effect of microstructures on functional nanostructures systematically, samples with tailored micro-cone heights and spatial periods were fabricated by tuning the laser scanning time and path, respectively. The influence of the micro-cone height and spatial period on the mechanical durability of the superhydrophobic metallic hierarchical surfaces was systematically studied. It was found that: (1) with the protection of micro-cones, both the tangential abrasion durability and the dynamic impact durability of the superhydrophobic metallic hierarchical surfaces were much higher than those of superhydrophobic metallic surfaces with single-scale nanostructures; (2) the micro-cone height played an important role in the improvement of the tangential abrasion durability; and (3) the micro-cone spatial period had a significant influence on the dynamic impact durability. The reason for the loss of superhydrophobicity was discussed from the viewpoint of both the destruction of the surface micro/nano morphology and the loss of the surface hydrophobic component. With an optimized micro-cone structure, tungsten-based superhydrophobic metallic hierarchical surfaces could withstand 70 abrasion cycles in linear abrasion tests, 28 min of solid particle impact in solid particle impact tests and 500 tape peeling cycles in tape peeling tests while the sliding angles remained less than 20°, which demonstrated exceptional comprehensive durability. The comprehensive durability that was achieved, in particular the dynamic impact durability and adhesive durability, are among the best published results to the best of our knowledge. This research reveals the mechanism whereby the microstructures effectively protected the functional nanostructures to achieve high durability of the superhydrophobic surfaces, as occurs in nature. Our approach is promising for enhancing the durability of superhydrophobic surfaces for practical applications.

## Conflicts of interest

There are no conflicts to declare.

## Supplementary Material

RA-008-C7RA13496G-s001
