# Supplementary material for: Comprehensively durable superhydrophobic metallic hierarchical surfaces via tunable micro-cone design to protect functional nanostructures
Source: RSC Adv. 2018 Feb 12;8(12):6733–44. doi: 10.1039/c7ra13496g (PMC9078309; doi:10.1039/c7ra13496g)
Supplement: RA-008-C7RA13496G-s001 [file RA-008-C7RA13496G-s001.pdf]

Electronic Supplementary Material (ESI) for RSC Advances.
This journal is © The Royal Society of Chemistry 2018

Electronic Supplementary Information

Comprehensively durable superhydrophobic metallic hierarchical surfaces via tunable micro-cone design to protect functional nanostructures

*Jinpeng Han, Mingyong Cai, Yi Lin, Weijian Liu, Xiao Luo, Hongjun Zhang, Kaiyang Wang, Minlin Zhong**

Laser Materials Processing Research Center, School of Materials Science and Engineering, Tsinghua University, Beijing 100084, P. R. China

*E-mail: zhml@tsinghua.edu.cn

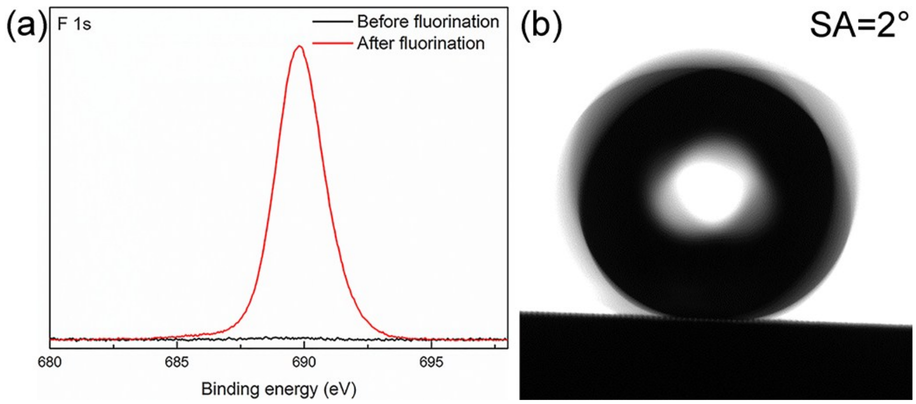

Fig. S1 Characterization of the Cu-based superhydrophobic hierarchical surfaces. (a) XPS spectra of F 1s. (b) Sliding angle with a water drop of 5 μL.

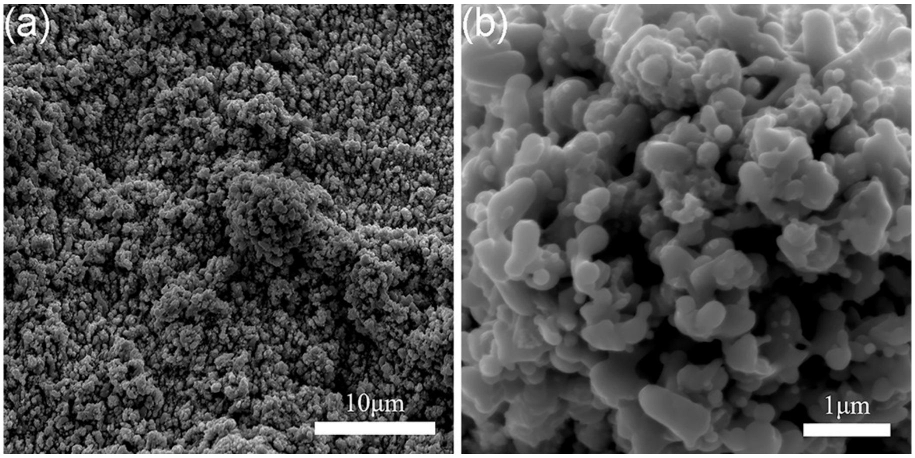

Fig. S2 SEM images of Sample NS in different magnifications.

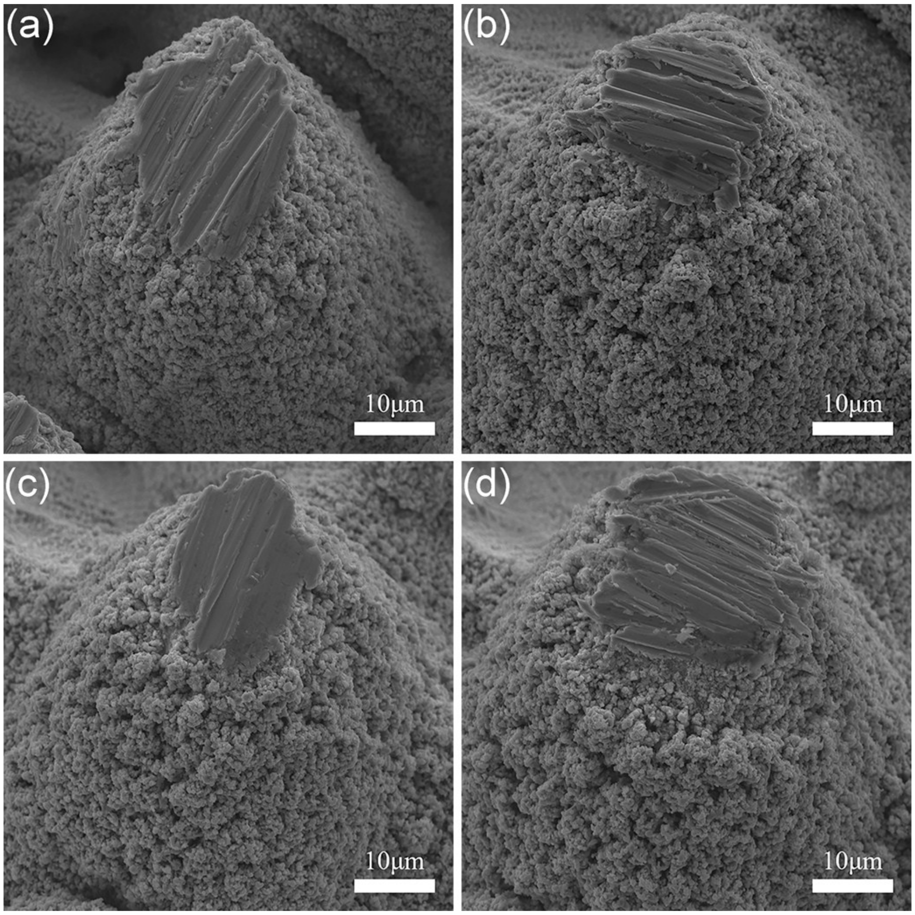

Fig. S3 SEM images of (a) Sample D60, (b) Sample D80, (c) Sample D100 and (d) Sample D120 after 15 abrasion cycles.

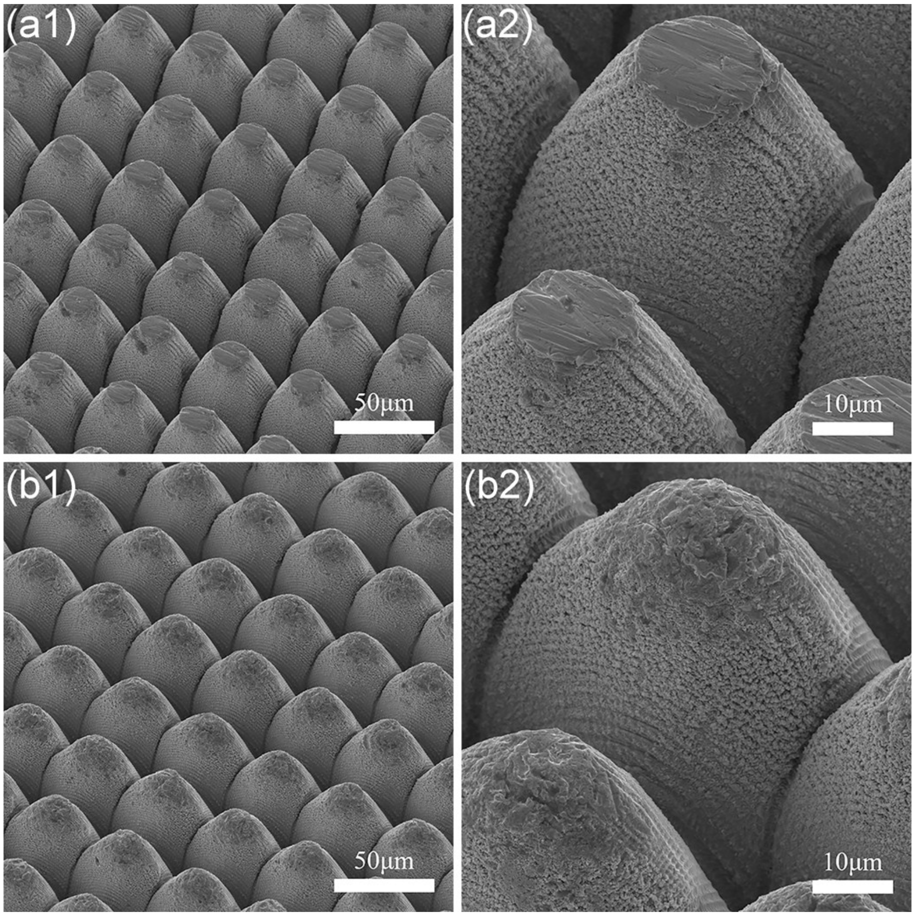

Fig. S4 SEM images of Sample W (a) after 100 abrasion cycles, (b) after 60 min of solid particle impacting.

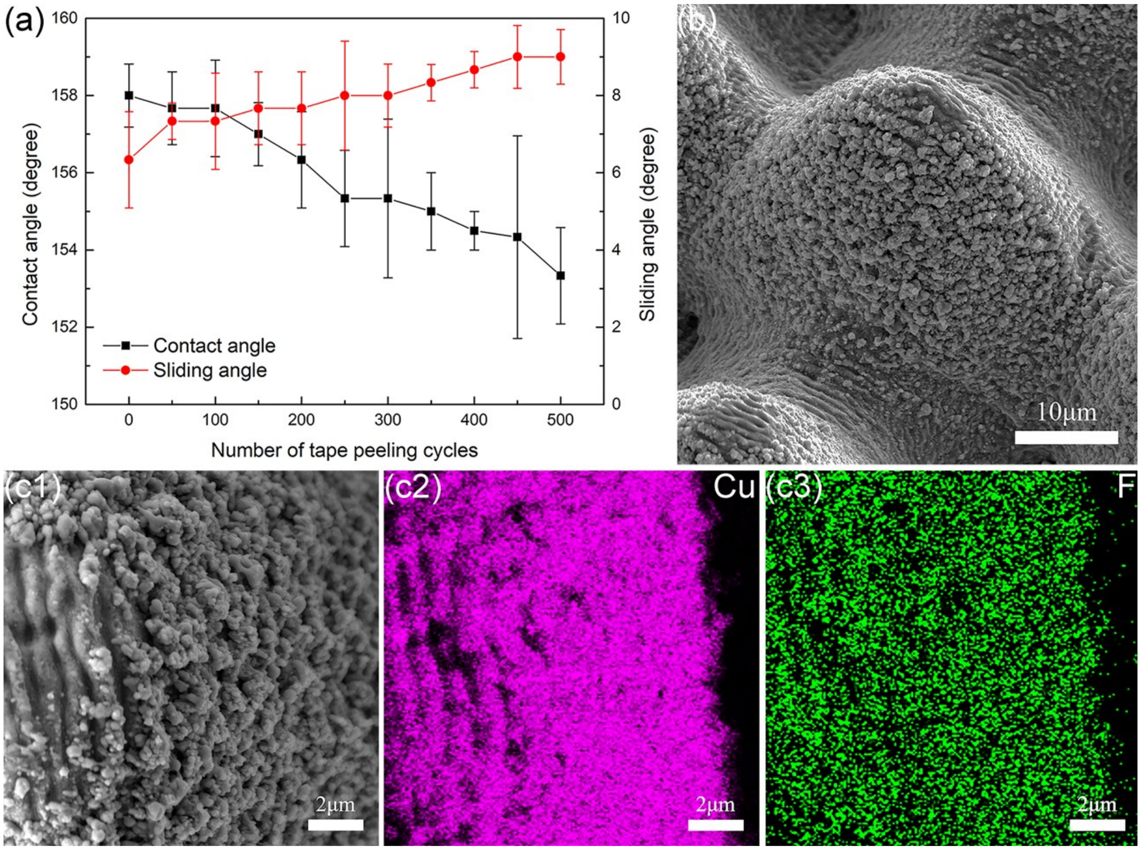

Fig. S5 Adhesive durability of Sample H25. (a) Contact angle and sliding angle as a function of number of tape peeling cycles. (b) SEM images of Sample H25 after 500 tape peeling cycles. (c) EDS mapping analysis of Sample H25 after 500 tape peeling cycles.

Table S1 Literature review of durable superhydrophobic surfaces with their materials, durability test conditions and wettability after durability tests. The durable performances of Sample W are also listed for comparison. Explanations of symbols are as follows. P: pressure, D: abrasion distance, N_A: number of abrasion cycles, Φ: diameter of solid particles, H: release height, V: release rate, T: time of impacting, M: mass of solid particles, N_I: number of solid particle impact cycles, F: adhesion strength of tape, N_P: number of tape peeling cycles.

Durability	Material	Durability Test Conditions	Wettability after Test	Ref
tangential abrasion durability	steel	400 # sandpaper, P=16 kPa, D=1.1 m	CA=152°, SA=8°	4
	aluminum	800 # sandpaper, P=0.98 kPa, D=1 m	CA=154°	8
	steel	260 # sandpaper, P=12.5 kPa, D=2.5 m	CA=153°, SA=10°	11
	copper	800 # sandpaper, P=4.8 kPa, D=1 m	CA=152°, SA=15°	30
	copper	800 # sandpaper, P=1.3 kPa, D=0.3 m	CA=138°, SA=22°	31
	fabric	1200 # sandpaper, P=13 kPa, N_A=30	CA=153°, SA=18°	34
	aluminum	1μm grade abrasive film, P=0.5 kPa, D=0.13 m	CA=155°, SA=9°	47
	tungsten	1000 # sandpaper, P=1.2 kPa, D=14 m	CA=155°, SA=19.7°	This paper
dynamic impact durability	steel	Φ=100~300 μm, H=10 cm, V=120 g min^{-1}, T=100 s	CA=150°	32
	polymer	Φ=200 μm, H=15 cm, T=5 min	CA=150°	35
	polymer	Φ=100~300 μm, H=40 cm, V=20 g min^{-1}, T=30 s	CA>150°, SA<10°	36
	polymer	Φ=100~300 μm, H=40 cm, M=50 g, N_I=20	CA=158°, CAH=23°	37
	silica	Φ=100~300 μm, H=30 cm, V=40 g min^{-1}, T=1 min	CA=170°	38
	aluminum	Φ=100~300 μm, H=30 cm, M=10 g, N_I=5	CA=150°, SA=16°	39
	silica	Φ=100~300 μm, H=30 cm, N_I=1	CA>150°, SA<10°	44
	silica	Φ=100~300 μm, H=25 cm, T=5 min	CA>150°, SA<10°	46
	tungsten	Φ=100~300 μm, H=25 cm, V=10 g min^{-1}, T=28 min	CA=155°, SA=19°	This paper
adhesive durability	silica	F=440 N m^{-1}, N_P=40	CA=157°	38
	aluminum	Scotch tape, N_P=10	CA=158°	40
	polymer	aluminum duct tape, N_P=4	CA=157°	41
	silica	double-sided tape, N_P=2	CA=159°	42
	polymer	F=820 N m^{-1}, N_P=12	CA=158°, CAH=9°	43
	silica	double-sided tape, N_P=1	CA>150°, SA<10°	44
	tungsten	F=710 N m^{-1}, N_P=500	CA=157°, SA=7.3°	This paper
